# Retrospective Analysis of Risk Factors in Geriatric Hip Fracture Patients Predictive of Surgical Intensive Care Unit Admission

**DOI:** 10.7759/cureus.60993

**Published:** 2024-05-24

**Authors:** Gerardo Trejo, Aiza Zia, Catherine Caronia, Abenamar Arrillaga, John Cuellar, Toyya A Pujol, Heather Reens, Florence LeFevre, Theresa Drucker, Sarah Eckardt, Randeep S Jawa, Patricia A Eckardt

**Affiliations:** 1 Family Medicine, Good Samaritan University Hospital, West Islip, USA; 2 Trauma, Good Samaritan University Hospital, West Islip, USA; 3 Pediatrics, Good Samaritan University Hospital, West Islip, USA; 4 Trauma/Surgical Critical Care, Good Samaritan University Hospital, West Islip, USA; 5 Orthopedic Surgery, Good Samaritan University Hospital, West Islip, USA; 6 Statistics, RAND Corporation, Cambridge, USA; 7 Nursing, Molloy University, Rockville Centre, USA; 8 Clinical Professional Development, North Shore University Hospital, Manhasset, USA; 9 Performance Improvement, Huntington Hospital, Northwell Health, Huntington, USA; 10 Division of Trauma Surgery, Stony Brook Medicine, Stony Brook, USA; 11 Nursing, Good Samaritan University Hospital, West Islip, USA

**Keywords:** geriatric, hip fracture, morbidity and mortality, surgical intensive care unit (sicu), geriatric hip fracture

## Abstract

Introduction: Although numerous risk factors and prediction models affecting morbidity and mortality in geriatric hip fracture patients have been previously identified, there are scant published data on predictors for perioperative Surgical Intensive Care Unit (SICU) admission in this patient population. Determining if a patient will need an SICU admission would not only allow for the appropriate allocation of resources and personnel but also permit targeted clinical management of these patients with the goal of improving morbidity and mortality outcomes. The purpose of this study was to identify specific risk factors predictive of SICU admission in a population of geriatric hip fracture patients. Unlike previous studies which have investigated predominantly demographic, comorbidity, and laboratory data, the present study also considered a frailty index and length of time from injury to presentation in the Emergency Department (ED).

Methods: A total of 501 geriatric hip fracture patients admitted to a Level 1 trauma center were included in this retrospective, single-center, quantitative study from January 1, 2019, to December 31, 2022. Using a logistical regression analysis, more than 25 different variables were included in the regression model to identify values predictive of SICU admission. Predictive models of planned versus unplanned SICU admissions were also estimated. The discriminative ability of variables in the final models to predict SICU admission was assessed with receiver operating characteristic curves’ area under the curve estimates.

Results: Frailty, serum lactate > 2, and presentation to the ED > 12 hours after injury were significant predictors of SICU admission overall (P = 0.03, 0.038, and 0.05 respectively). Additionally, the predictive model for planned SICU admission had no common significant predictors with unplanned SICU admission. Planned SICU admission significant predictors included an Injury Severity Score (ISS) of 15 and greater, a higher total serum protein, serum sodium <135, systolic blood pressure (BP) under 100, increased heart rate on admission to ED, thrombocytopenia (<120), and higher Anesthesia Society Association physical status classification (ASA) score (P = 0.007, 0.04, 0.05, 0.002, 0.041, 0.05, and 0.005 respectively). Each SICU prediction model (overall, planned, and unplanned) demonstrated sufficient discriminative ability with the area under the curve (AUC) values of 0.869, 0.601, and 0.866 respectively. Finally, mean hospital Length of Stay (LOS) and mortality were increased in SICU admissions when compared to non-SICU admissions.

Conclusion: Of the three risk factors predictive of SICU admission identified in this study, two have not been extensively studied previously in this patient population. Frailty has been associated with increased mortality and postoperative complications in hip fracture patients, but this is the first study to date to use a novel frailty index specifically designed and validated for use in hip fracture patients. The other risk factor, time from injury to presentation to the ED serves as an indicator for time a hip fracture patient spent without receiving medical attention. This risk factor has not been investigated heavily in the past as a predictor of SICU admissions in this patient population.

## Introduction

Approximately 1.6 million osteoporotic hip fractures occur each year around the world, and although the incidence of hip fractures may have decreased over the last 20 years [1], the overall number of fractures has increased substantially in developed nations. Certain models predict that by the year 2040, the United States (US) will see upwards of 840,000 osteoporotic hip fractures per year [2,3]. Overall, outcomes for geriatric patients who present to the hospital with hip fractures can vary greatly depending on numerous factors including age [4], sex [5], co-morbidities [6], frailty scores [7], and type of fracture [8].

While some surgical patients follow an uncomplicated postoperative period with an eventual return to prior functional status, some patients suffer from complications ultimately requiring critical care management with a subsequent increased risk of mortality [9]. Determining which hip fracture patients are at increased risk for Surgical Intensive Care Unit (SICU) admission is a subject of high investigative interest, particularly in an era of economic burden and resource allocation. Identifying which patients are more likely to require SICU admission early on would help prevent unplanned Intensive Care Unit (ICU) admissions and allow appropriate targeted resource allocation to help reduce morbidity and mortality in those hip fracture patients found to be at higher risk.

Although there exist many different scoring systems internationally validated to predict morbidity and mortality for orthopedic surgery patients, the most common predictive models include the Charlson Comorbidity Index (CCI) [10], the Estimation of Physiologic Ability and Surgical Stress (E-PASS) [11], the Orthopedic Physiologic Operative Severity Score for the enUmeration of Mortality and Morbidity (O-PPOSUM) [12], the risk model utilized by Jiang et al. [13], and the Nottingham Hip Fracture Score (NHFS) [14]. Of the models listed above, only the NHFS and Jiang models were specifically designed for mortality prediction in the hip fracture population. The Orthopedic Hip Frailty Score (OFS) is a newer frailty index designed for the assessment of mortality prediction specifically in orthopedic hip fracture patients [15]. Unlike the majority of the other indices listed which rely heavily on comorbidity, laboratory, and operative data, the OFS is calculated using only five variables, including institutionalization, non-independent functional status, and a history of malignancy, which have all been largely accepted as markers for frailty [16].

Even though the majority of the predictive indices listed above are particularly useful in predicting mortality in hip fracture patients, only a handful of investigators have worked to identify similar risk factors predictive of SICU admission in this geriatric hip fracture population [17]. The proposed study at our institution aims to identify risk factors predictive of SICU admission from a large pool of clinical variables including a novel frailty index and temporal Emergency Department (ED) presentation data (i.e., the time elapsed from injury to presentation in the ED).

## Materials and methods

Study design

This retrospective, single-center, quantitative study was conducted by the Department of Trauma Surgery in conjunction with the Department of Orthopedic Surgery at a Level 1 trauma center in Long Island, New York. Of note, in accordance with the US federal Emergency Medical Treatment & Labor Act (EMTALA) guidelines specific to New York State, all patients arriving via ambulance must be brought to the nearest emergency room, regardless of the institution’s trauma center level for a medical screening examination. Although the institution utilized for the study was a Level 1 trauma center, it must be understood that patients were brought to the institution because of their proximity to the location, as opposed to requiring services that only a Level 1 trauma center could provide, such as treatment of high-impact fractures and polytrauma patients [18,19].

Patients were included in the study if they were aged 75 years or greater with an International Classification of Diseases-10 (ICD-10) code for hip fracture admitted to the trauma center during the time period from January 1, 2019, through December 31, 2022. Exclusion criteria were patients younger than 75 years of age and patients admitted for a hip fracture outside of the sampling time frame. The rationale for the inclusion of patients 75 years and older was based on recent literature on the impact of frailty and risk assessment of older orthopedic patients for postoperative complications [20-23].

Data collection

After approval from the institutional review board (IRB) for research with human subjects (IRB#: 2022.09.07), data from patients who met the inclusion criteria were exported from the electronic health record and stored as .csv files in Research Electronic Data Capture (REDCap) (Vanderbilt University Medical Center, Nashville, Tennessee, USA) for primary research record source. Data was then exported into Microsoft Excel (Microsoft Corporation, Redmond, Washington, USA) for data recording and transformation.

Outcomes and clinical variables

The primary outcome measure being evaluated in this patient population was an admission to the SICU. The decision to admit a patient to SICU as a planned admission is made by the trauma surgeon and can be based on prior patient medical history or presenting clinical factors such as tachycardia or high serum lactate on ED presentation. The total number of patients included in the study was 501 and this cohort was divided into two groups, those who required an SICU admission and those who did not. The predictive model derived for SICU admission (overall admission) was based on these two groups. The SICU admission group was then further subdivided into two groups: the first group consisted of patients not initially admitted to SICU but transferred to the SICU postoperatively by the trauma surgeon managing their care (unplanned group); the second group consisted of patients initially admitted to SICU directly from the ED (planned group) based on the trauma surgeon’s assessment. Predictive models for SICU admission were also run for comparison between these two groups. Additional outcome measures evaluated in this study included hospital Length of Stay (LOS) and overall mortality rates compared between the patients admitted to the SICU versus those not admitted to the SICU. Lastly, the outcomes of LOS and SICU LOS were also compared between the unplanned and planned SICU admission groups.

The clinical and demographic risk factors being investigated in this study were chosen because they had previously been associated with the risk of mortality or SICU admission for hip fracture patients as described in the background above. Demographic variables collected from Electronic Medical Record (EMR) data included sex, age, and Body Mass Index (BMI). Clinical characteristics included time from injury to ED presentation, trauma activation level, mechanism of injury, ED disposition, admitting service, ED arrival Glasgow Coma Scale (GCS) score, Injury Severity Score (ISS), Anesthesia Society Association physical status classification (ASA), vital signs, and common admission laboratory values such as lactic acid and those included in the comprehensive metabolic panel (CMP), and the complete blood cell count (CBC) results. Derived variables such as frailty (taken from the OFS) and the updated CCI were also calculated from the EMR data collected for inclusion in the study.

Statistical analysis

The statistical software Statistical Package for Social Sciences (SPSS), version 29.0 (IBM Corp., Armonk, NY), and Stata/Special Edition (SE) 18 (StataCorp LLC, College Station, Texas, USA) were used to perform descriptive, and inferential analyses. Independent sample t-tests were used to compare the means between two groups (SICU admission or non-SICU admission) on the continuous variables. Chi-square (χ2) tests were used to compare proportional differences between the two groups on the nominal variables. Logistic regression models were generated using the backward stepwise (Wald) method to elucidate the risk factors for SICU admission in the geriatric hip fracture population. Inferential analyses used a two-tailed approach, and 0.05 was chosen as the a priori critical alpha value. Power was sufficient with more than ten cases per independent variable in the model [24]. Additionally, the discriminative ability of the risk factors in the final model was assessed individually and as a combined single variable using the area under the curve (AUC) analysis from the receiver operating characteristic (ROC) curve. ROC curves were conducted in two separate analyses for the planned SICU admission risk factors to account for the inverse relationship of one of the risk factors with the probability of SICU admission [25]. The combined single variable was derived from regressing postoperative SICU admission on the variables in the final risk factor models for SICU admission. Lastly, secondary outcomes of hospital LOS and mortality were compared between groups of SICU admissions versus non-SICU admissions, and planned versus unplanned SICU admissions, respectively.

## Results

Among the 501 patients included in this cohort, 62 (12%) experienced an SICU admission (Table 1), 17 were planned and 45 were unplanned (Table 2). Most of the sample was female (74%), with a mean age of 86.1 years for the total sample. The majority (98.4%) of the patients in the sample’s mechanism of injury was a fall (98.6% and 96.8% in the non-SICU admissions and SICU admission groups respectively). The results of the univariate analysis demonstrated some differences between those patients admitted to the SICU and those who were not. Patients admitted to the SICU had significantly lower oxygen saturation (not admitted to SICU, 96.55%; admitted to SICU, 95.79%), higher updated CCI (uCCI; not admitted to SICU, 0.91%; admitted to SICU, 08.06%), ASA (not admitted to SICU, 01.82%; admitted to SICU, 11.29%), and ISS values (not admitted to SICU, 9.51, SD=1.68; admitted to SICU, 10.73, SD=04.62) (P = 0.05; P < 0.001; P < 0.001; and P = 0.04 respectively). The patients admitted to the SICU also had a significantly longer hospital LOS (9.31, SD=06.94, P <0.01) than those not admitted to the SICU (4.81, SD=2.38, P <0.01). These factors, however, were not significant in the multivariate analyses.

**Table 1:** Sample Characteristics for Patients Admitted to SICU and Patients Not Admitted to SICU (n=501). ^a^Mean, SD; ^b^Frequency, percentage; ^c^Independent sample t-tests were conducted for continuous variable estimates, crosstabulation chi-square estimates were conducted for categorical data (except when expected count < 5 in a cell Fisher’s exact statistic was estimated); ^d^Frailty was defined using Orthopedic Hip Frailty Score >=2; ^e^The chosen critical alpha level for all significance tests was 0.05 or less. *p values significant at 0.05 or <; **p values significant at 0.01 or <; ***p values significant at 0.01 or <. SICU, Surgical Intensive Care Unit; MOI, Mechanism of Injury; ED, Emergency Department; GCS, Glasgow Coma Score; SBP, Systolic Blood Pressure; BP, Blood Pressure; HR, Heart Rate; uCCI, updated Charlson Comorbidity Index; ASA, Anesthesia Society Association physical status classification; BMI, Body Mass Index; OR, Operating Room; ISS, Injury Severity Score; LOS, Length of Stay; ICU, Intensive Care Unit; HGB, Hemoglobin; WBC, White Blood Cell.

Variable	No SICU Admit (n=439)	Admitted to SICU (n=62)	p-value^e^
MOI = Fall	433 (98.60%)	60 (96.80%)	0.28
Age^a^	86.29 (SD=5.86)	85.79 (SD=04.85)	0.52
Sex			--
-Male^b^	113 (25.74%)	17 (27.42%)	0.78
-Female^b^	326 (74.26%)	17 (27.42%)	0.18
ED Admission Braden Score^a^	16.28 (SD=2.59)	15.91 (SD=03.56)	0.50
ED Arrival GCS^a^	14.69 (SD=0.72)	14.75 (SD=00.79)	0.57
ED Arrival SBP^a^	148.32 (SD=25.19)	143.56 (SD=27.67)	0.17
-SBP <100	8 (1.82%)	3 (4.84%)	1.14
ED Arrival HR^a^	79.62 (SD=15.19)	81.65 (SD=19.56)	0.35
ED Arrival Temp F^a^	97.91 (SD=0.72)	97.83 (SD=00.64)	0.45
ED Arrival Oxygen Saturation^a^	96.55 (SD=2.79)	95.79 (SD=03.42)	0.05*
uCCI			<0.001^c***^
-CCI 0^b^	414 (94.31%)	48 (77.42%)	--
-CCI 1^b^	3 (0.68%)	0 (0.0%)	--
-CCI 2^b^	14 (3.19%)	8 (12.90%)	--
-CCI 3^b^	4 (0.91%)	1 (01.61%)	--
-CCI 4^b^	4 (0.91%)	5 (08.06%)	--
ASA			<0.001***
-ASA 1^b^	354 (80.64%)	42 (67.74%)	--
-ASA 2^b^	47 (10.71%)	11 (17.74%)	--
-ASA 3^b^	8 (01.82%)	7 (11.29%)	--
Orthopedic Hip Frailty^b,d^	192 (43.74%)	26 (41.94%)	0.80
Albumin^a^	3.49 (SD=0.42)	3.51 (SD=00.36)	0.79
BMI^a^	24.31 (SD=4.72)	23.98 (SD=05.46)	0.61
>12 Hour From Injury^b^	79 (18.00%)	11 (17.74%)	0.93
Time to 1st OR Visit from ED arrival (in minutes)	1533.46 (SD=978.2)	2042.19 (SD=1564.72)	<0.02*
ISS^a^	9.51 (SD=1.68)	10.73 (SD=04.62)	0.04*
-ISS >=15^b^	8 (1.82%)	6 (09.68%)	<0.001***
Hospital LOS^a^	4.81 (SD=2.38)	9.31 (SD=06.94)	<0.01**
ICU LOS days^a^	NA	5.3 (SD=03.88)	NA
HGB^a^	11.92 (SD=1.7)	11.92 (SD=02.38)	0.99
Protein Total^a^	6.93 (SD=0.61)	6.78 (SD=00.74)	0.26
-Low Protein^b^	9 (02.05%)	3 (04.84%)	0.08
Sodium^a^	139.22 (SD=3.22)	138.81 (SD=03.32)	0.51
-Hypokalemia^b^	32 (07.29%)	4 (06.45%)	0.99
Mean Platelet Volume^a^	10.28 (SD=0.94)	10.33 (SD=00.80)	0.70
-Thrombocytopenia^b^	18 (4.10%)	3 (04.84%)	0.68
WBC^a^	11.02 (SD=3.97)	10.7 (SD=04.28)	0.61

**Table 2:** Covariates by Patients Planned to be Admitted to SICU and Unplanned SICU Admissions. ^a^Mean, SD; ^b^Frequency, percentage; ^c^Independent sample t-tests were conducted for continuous variable estimates, crosstabulation chi-square estimates were conducted for categorical data (except when expected count < 5 in a cell Fisher’s exact statistic was estimated); ^d^Frailty was defined using Orthopedic Hip Frailty Score >=2; ^e^The chosen critical alpha level for all significance tests was 0.05 or less. *p values significant at 0.05 or <; ***p values significant at 0.01 or <. SICU, Surgical Intensive Care Unit; MOI, Mechanism of Injury; ED, Emergency Department; GCS, Glasgow Coma Score; SBP, Systolic Blood Pressure; BP, Blood Pressure; HR, Heart Rate; CCI, Charlson Comorbidity Index; ASA, Anesthesia Society Association physical status classification; BMI, Body Mass Index; OR, Operating Room; ISS, Injury Severity Score; LOS, Length of Stay; ICU, Intensive Care Unit; HGB, Hemoglobin; WBC, White Blood Cell.

Variable	Planned SICU Admit (n=17)	Unplanned SICU Admit (n=45)	p-value^e^
Age^a^	86.24 (SD=5.02)	85.62 (SD=4.83)	0.66
Sex			0.53^c^
-Male^b^	5 (29.41%)	12 (26.67%)	--
-Female^b^	12 (70.59%)	33 (73.33%)	--
ED Admission Braden Score^a^	16.08 (SD=3.9)	15.85 (SD=3.48)	0.82
ED Arrival GCS^a^	14.56 (SD=0.81)	14.82 (SD=0.79)	0.26
ED Arrival SBP^a^	132.88 (SD=34.71)	147.6 (SD=23.71)	0.061
-SBP <100	3 (17.65%)	0 (0.00%)	0.02^c*^
ED Arrival HR^a^	83.06 (SD=27)	81.11 (SD=16.25)	0.78
ED Arrival Temp F^a^	97.76 (SD=0.75)	97.86 (SD=0.6)	0.62
ED Arrival Oxygen Saturation^a^	95.41 (SD=3.61)	95.93 (SD=3.37)	0.61
uCCI			0.43
-CCI 0^b^	14 (82.35%)	34 (75.56%)	--
-CCI 1^b^			--
-CCI 2^b^	3 (17.65%)	5 (11.11%)	--
-CCI 3^b^		1 (2.22%)	--
-CCI 4^b^		5 (11.11%)	--
ASA			0.51
-ASA 1^b^	13 (76.47%)	29 (64.44%)	--
-ASA 2^b^	2 (11.76%)	9 (20.00%)	--
-ASA 3^b^	1 (05.88%)	6 (13.33%)	--
Orthopedic Hip Frailty ^b,d^	8 (47.06%)	18 (40.00%)	0.75^c^
Albumin^a^	3.6 (SD=0.37)	3.48 (SD=0.37)	0.26
BMI^a^	23.3 (SD=4.68)	24.22 (SD=5.75)	0.56
>12 Hour From Injury^b^	2 (11.76%)	9 (20.00%)	0.71^c^
Time to 1st OR Visit (in minutes)	2594.14 (SD=1574.8)	1866.57 (SD=1537.84)	0.10
ISS^a^	14.35 (SD=7.74)	9.36 (SD=0.88)	<0.001***
-ISS >=15^b^	6 (35.29%)		<0.001^c***^
Hospital LOS^a^	8.29 (SD=6.01)	9.69 (SD=7.29)	0.49
ICU LOS days^a^	6 (SD=4.24)	5.04 (SD=3.77)	0.39
HGB^a^	11.88 (SD=2.88)	11.93 (SD=2.2)	0.94
Protein Total^a^	6.67 (0.75)	6.81 (0.76)	0.52
-Low Protein^b^	1 (05.88%)	2 (04.44%)	0.86^c^
Sodium^a^	138.13 (SD=4.02)	139.04 (SD=3.11)	0.35
-Hypokalemia^b^		4 (8.89%)	0.55^c^
Mean Platelet Volume^a^	10.56 (SD=0.77)	10.24 (SD=0.81)	0.17
-Thrombocytopenia^b^	2 (11.76%)	1 (02.22%)	0.18^c^
WBC^a^	10.68 (SD=5.2)	10.7 (SD=3.96)	0.98

This model demonstrated overall significance in predicting SICU admission (P = 0.001), explained sufficient variation in the outcome of interest (Nagelkerke R2 =.517), and had an overall correct prediction percentage of 84.0 (Table 3). The model for unplanned SICU admission, which represented 73% of all SICU admissions, included the three risk factors identified for overall SICU admission, and one more risk factor, time to Operating Room (OR) in minutes (Tables 3, 4).

**Table 3:** Logistic Regression Models for Identifying Factors for Patients With Hip Fracture Being Admitted to the SICU. ^a^Statistical significance a priori one-tailed 0.05 or less level; ^b^Frail was defined using the Orthopedic Hip Frailty score (greater than or equal to 2 is frail); ^c^Nagelkerke estimate for Pseudo R^2^ statistics. CI, Confidence Interval; SICU, Surgical Intensive Care Unit; ED, Emergency Department; BP, Blood Pressure; ASA, Anesthesia Society Association physical status classification; OR, Operating Room; ISS, Injury Severity Score.

	Odds Ratio	95% CI	P value^a^
Model 1. Admission to SICU (pooled)			
Frail^b^	14.64	0.87, 247.4	0.03
Serum lactate > 2	13.40	0.74, 241.2	0.038
Presented to ED > 12 hours after injury	7.50	0.60, 93.2	0.05
Model significance	0.001	--	--
Pseudo R^2 c^	.517	--	--
Hosmer and Lemeshow	.738	--	--
Percentage correct	84.0	--	--
Model 2. Planned admission to SICU			
ISS 15 or greater	22.01	1.56, 309.12	0.007
Total serum protein	0.32	0.08, 1.27	0.04
Serum sodium < 135	16.97	1.73, 165.6	0.05
Systolic BP < 100 on arrival to ED	142.41	4.4, 4598.3	0.002
Heart rate on arrival to ED	1.06	0.99, 1.14	0.041
ASA score	1.98	0.44, 8.8	0.05
Thrombocytopenia	22.07	1.81, 268.7	0.005
Model significance	0.001	--	--
Pseudo R^2^	.504	--	--
Hosmer and Lemeshow	.921	--	--
Percentage correct	96.3	--	--
Model 3. Unplanned admission to SICU			
Frail^c^	15.03	0.58, 387.8	0.05
Serum lactate > 2	24.47	0.60, 998.5	0.04
Presented to ED > 12 hours after injury	17.05	0.60, 481.5	0.04
Time to first OR visit in minutes	1.80	0.11, 1.98	0.08
Model significance	0.048	--	--
Pseudo R^2^	.510	--	--
Hosmer and Lemeshow	.565	--	--
Percentage correct	82.6	--	--

**Table 4:** Risk Factors for Unplanned SICU Admission. SICU, Surgical Intensive Care Unit; CI, Confidence Interval; ED, Emergency Department; OFS, Orthopedic Frailty Score; OR, Operating Room.

Unplanned SICU Admission	Area Under the Curve	Standard Error	Lower Bound 95% CI	Upper Bound 95% CI
Presentation to ED > 12 hours after injury	0.723	0.127	0.474	0.972
Frailty (OFS >=2)	0.679	0.118	0.448	0.909
Lactate =>2	0.679	0.118	0.448	0.909
Time to OR in minutes	0.616	0.145	0.333	0.899

**Figure 1:**
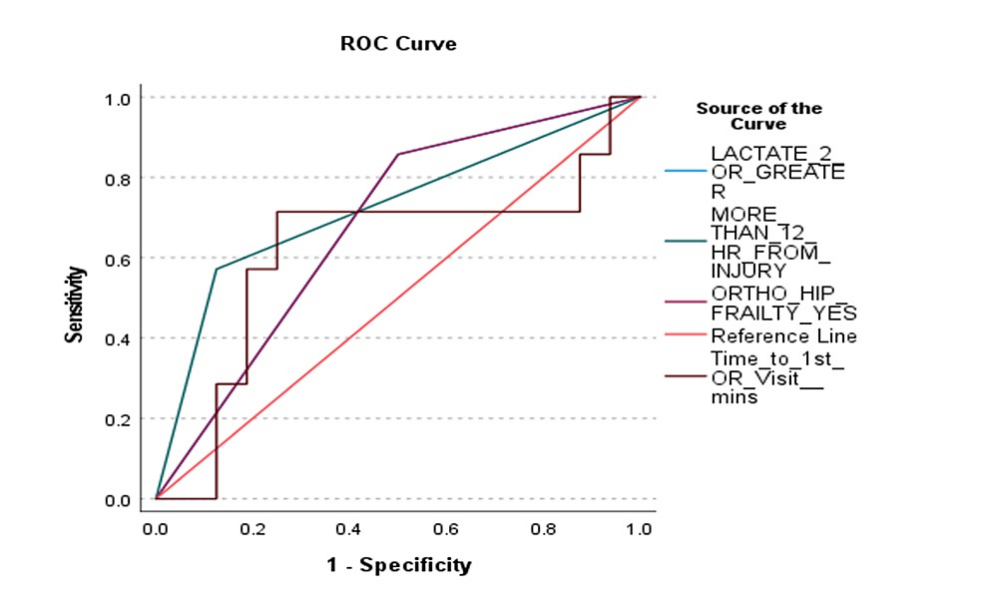
Receiver Operating Characteristic (ROC) Curve for Unplanned SICU Admission Risk Factors. Risk factors have a positive relationship with the probability of Surgical Intensive Care Unit (SICU) admission. There is at least one tie between positive and negative actual state groups. HR, Hour; OR, Operating Room.

**Figure 2:**
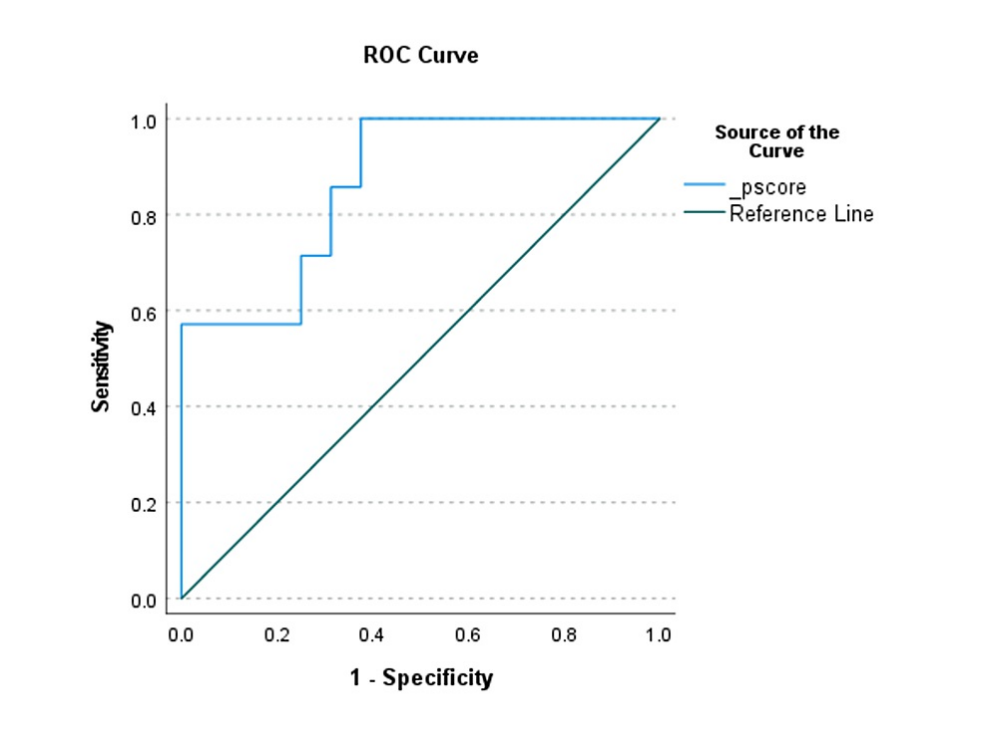
Receiver Operating Characteristics (ROC) Curve of Propensity Score of Predictors for Unplanned SICU Admissions. There is at least one tie between positive and negative actual state groups. SICU, Surgical Intensive Care Unit, Pscore, Propensity Score.

This model demonstrated overall significance in predicting SICU admission (P = 0.048), explained sufficient variation in the outcome of interest (Nagelkerke R2 =.510), and had an overall correct prediction percentage of 82.6. A range in the discriminative ability of the risk factors in the final model was found (Figures 1, 2).

The final model (Equation 1) for unplanned SICU admission included frailty (OFS >= 2), serum lactate greater than 2, presentation to the ED more than 12 hours after the injury occurred, and time to the OR.

Logit p = -7.48 + 2.71* frailty + 3.97*serum lactate > 2 + 2.84* presentation to ED > 12 hours after injury + 0.08*time to OR in minutes

Interestingly, the significant risk factors of planned ICU admission had no commonality with unplanned SICU admission predictors. The model demonstrated overall significance (P = 0.001), explained sufficient variation in the outcome of interest (Nagelkerke R2 =.504), with seven risk factors (Equation 2), and had an overall correct prediction percentage of 96.3 in the model (Table 3).

Logit p = -10.03 + 2.55*ISS=>15 + 6.87*ASA score + 2.16* total serum protein+ 5.32* Systolic BP < 100 on arrival to ED + 2.75* Serum sodium < 135 + 3.27* thrombocytopenia + 0.048* Heart rate on arrival to ED

**Figure 3:**
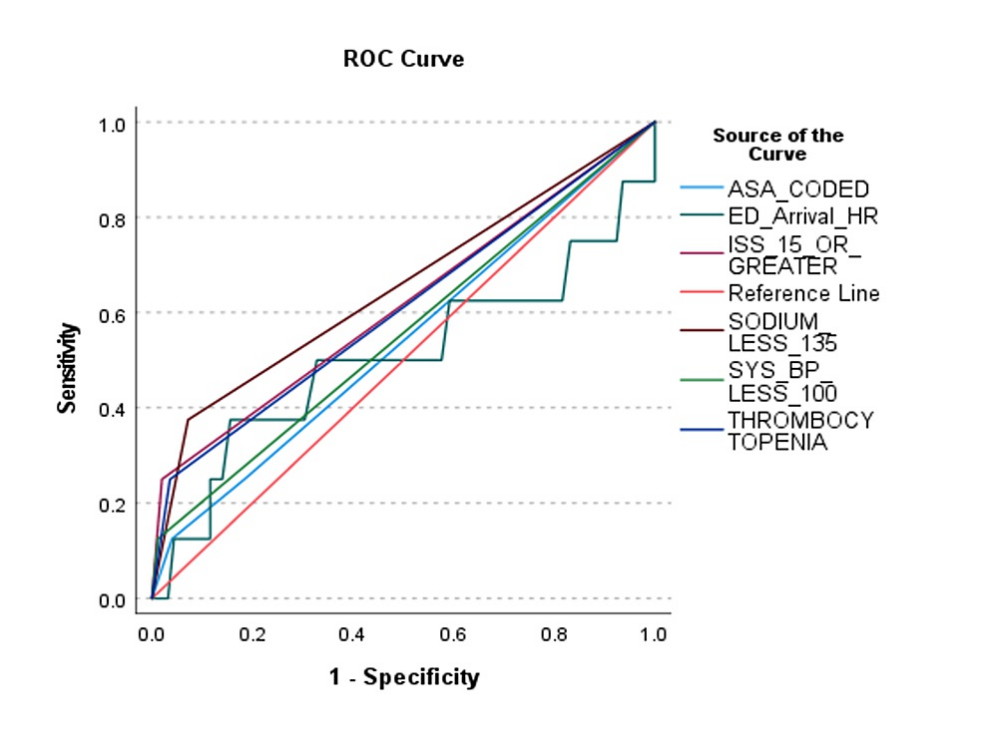
Receiver Operating Characteristic (ROC) Curve for Predictors That Have a Positive Relationship With SICU Admission. There is at least one tie between positive and negative actual state groups. SICU, Surgical Intensive Care Unit; ASA, Anesthesia Society Association physical status classification; ED, Emergency Department; HR, Heart Rate; ISS, Injury Severity Score; BP, Blood Pressure.

**Figure 4:**
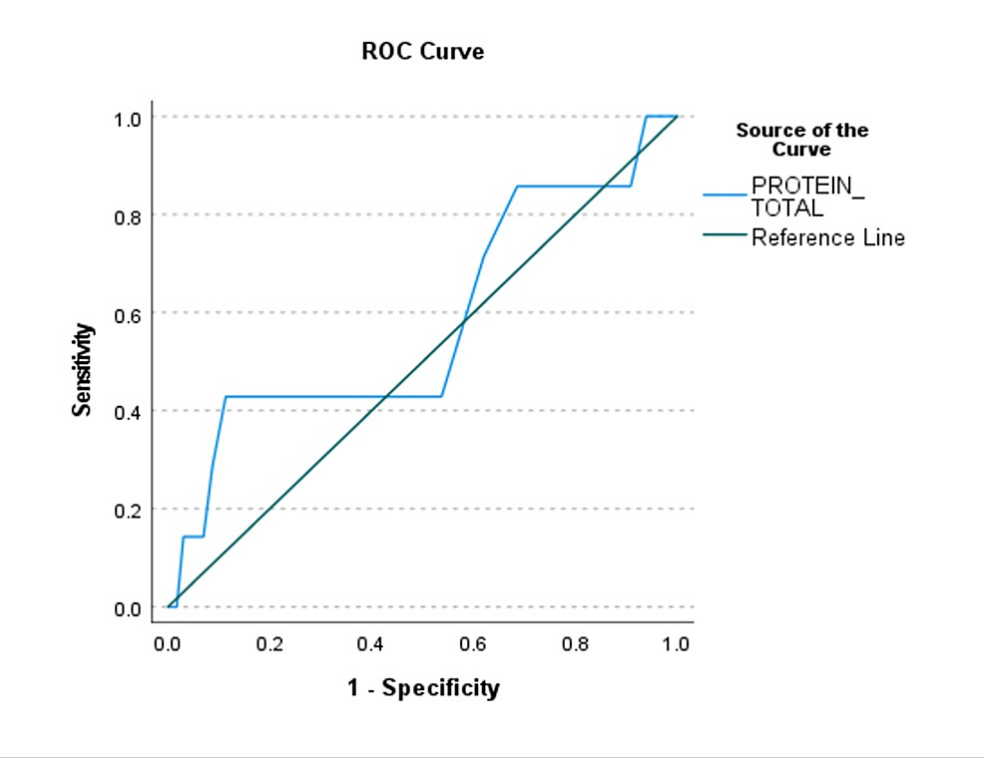
Receiver Operating Characteristic (ROC) Curve for the Predictor That Has an Inverse Relationship With Planned SICU Admission. There is at least one tie between positive and negative actual state groups. SICU, Surgical Intensive Care Unit.

**Figure 5:**
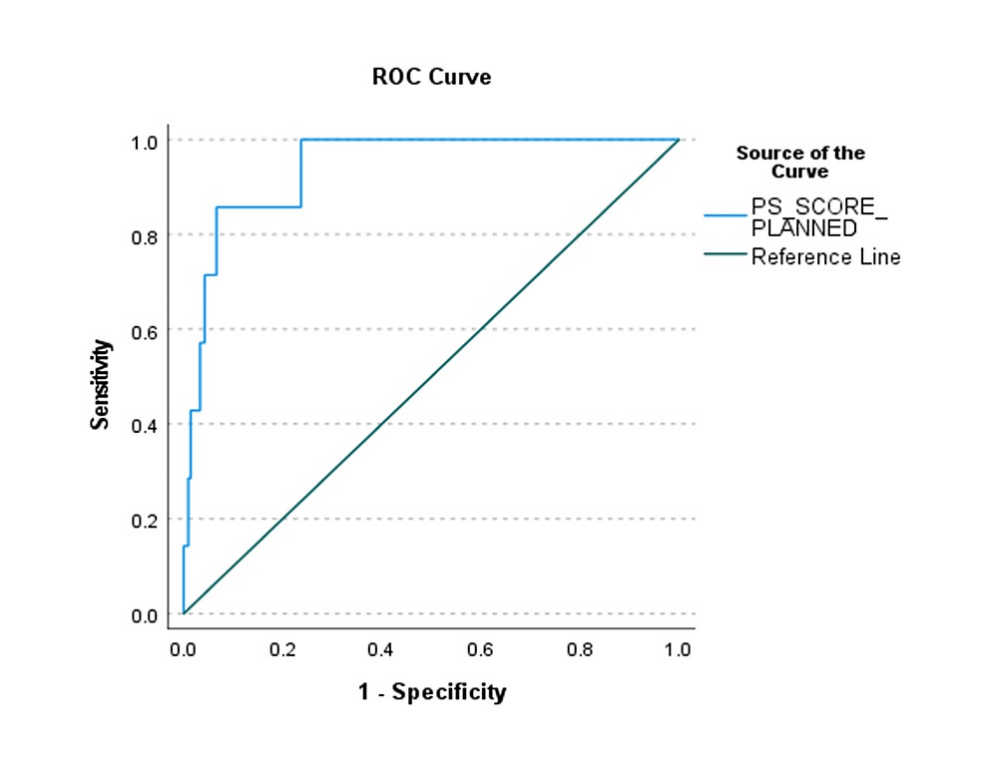
Receiver Operating Characteristic (ROC) Curve for Planned SICU Admission Propensity Score of Risk Factors. There is at least one tie between positive and negative actual state groups. SICU, Surgical Intensive Care Unit.

A range in the discriminative ability of the predictor variables in the final model was found (Figures 3-5) (Table 5).

**Table 5:** Risk Factors for Planned SICU Admission. SICU, Surgical Intensive Care Unit; CI, Confidence Interval; ISS, Injury Severity Score; ED, Emergency Department; BP, Blood Pressure; ASA, Anesthesia Society Association physical status classification.

Planned SICU Admission	Area Under the Curve	Standard Error	Lower Bound 95% CI	Upper Bound 95% CI
Serum sodium < 135	0.677	0.115	0.439	0.916
ISS =>15	0.634	0.117	0.388	0.880
Thrombocytopenia	0.607	0.116	0.380	0.834
Total serum Protein	0.580	0.124	0.338	0.823
ED Systolic BP < 100	0.569	0.112	0.330	0.808
ASA score	0.557	0.110	0.324	0.790
Heart Rate on Arrival to ED	0.505	0.130	0.289	0.760

Mean hospital LOS and mortality were significantly higher (Table 6) for patients admitted to SICU than non-SICU admissions (P < 0.001, P< 0.001 respectively). There was no significant difference in mean hospital LOS, SICU LOS, or mortality between planned and unplanned SICU admissions (P =0.485, P= 0.402, and P= 0.942 respectively). 

**Table 6:** Comparisons Between Groups of Length of Stay, Mortality, and SICU Length of Stay. ^a^Significance tests for continuous and categorical variables were two-tailed independent samples t-test, and a cross-tabulation two-tailed chi-square estimate with 1 degree of freedom (Fisher’s exact estimate for cells with expected value 5 or less), respectively; ^b^SD, Standard deviation; ^c^Percentage within group. SICU, Surgical Intensive Care Unit.

Outcome	Group				P value^a,b^
	Non-SICU admission	SICU admission			
		Pooled SICU admissions	Planned SICU admission	Unplanned SICU admission	
Mean Hospital Length of Stay in Days (SD)^b^	4.8 (2.4)	9.3 (6.9)	--	--	< .001
	--	--	8.3 (6.1)	9.7 (7.3)	.485
Mean SICU Length of Stay in Days (SD)^b^			6.0 (4.2)	5.0 (3.8)	.402
Mortality Count (%)^c^	5 (1.1%)	7 (11.3%)			< .001
			2 (11.8%)	5 (11.1%)	.942

## Discussion

Differences in patients admitted to SICU versus patients not admitted to SICU

As expected, the results of univariate analysis revealed significant differences in hip fracture patients admitted to the SICU when compared to those patients not admitted to the SICU. Not surprisingly, hip fracture patients admitted to the SICU had significantly lower oxygen saturations than those not admitted to the SICU. Although ICU admission guidelines vary greatly across the country, persistent hypoxemia or impending respiratory failure requiring respiratory support generally meets SICU admission criteria for our institution. The finding that patients admitted to the SICU were more likely to have lower oxygen saturations than those not in the SICU may be expected as persistent hypoxemia or impending respiratory failure requiring respiratory support generally meet SICU admission criteria for our institution.

Patients admitted to the SICU also had higher uCCI, ASA, and ISS values. The higher uCCI and ASA score findings are consistent with previous findings that hip fracture patients tend to be sicker at the time of admission, partly due to decompensation of comorbid illnesses and higher frailty scores than those patients with other traumatic injuries. Higher uCCI scores have been associated with higher postoperative complications, mortality [26,27] costs, and LOS [28]. The SICU-admitted patients’ higher ISS values as predictive of in-hospital complications require further exploration. Though our study had a smaller sample that included hip fracture patients only, these findings may align with a recent large national retrospective (n=40,800) study that found a high ISS was an independent predictor of in-hospital mortality from a ground level fall in geriatric patients with normal physiological parameters upon presentation to the trauma center [29].

Predictors of SICU admission

Hyperlactatemia was one of the risk factors predictive for SICU admission identified in this study. This finding was not surprising given previous extensive research demonstrating elevated serum lactate as a mortality predictor in patients with hip fractures [30]. Physiologically, hyperlactatemia in these patients is most often secondary to anaerobic metabolism from prolonged hypoperfusion or ischemia [31]. Elevated lactate, in general, can be a very useful marker for identifying the grade of biological injury in those patients who have suffered traumatic insults despite variances in physiological reserve and comorbidity status [32]. As such, lactate can also be used as a biochemical marker of injury severity in patients suffering from trauma [24]. Specifically in this study population, whose average age was 86.04, we included serum lactate as it has been found to be a marker of frailty and is not included in the OFS scale [33]. Worsening hyperlactatemia is indicative of a more severe injury, likely needing critical care management requiring SICU admission. In the present study, a normal lactate was defined as less than 2 mmol/L. Hyperlactatemia was defined as any value greater than 2 mmol/L. Although prior predictive studies have utilized different cutoff values for using lactate as a predictor for SICU admission, we were interested in using any values that deviated from normal, not just elevated values that might automatically trigger SICU admission criteria independently. For example, a geriatric hip fracture patient with a lactate of 8 mmol/L is most likely going to meet the criteria for ICU level of care, or at least merit an evaluation by a critical care team consultant for possible ICU admission. One of the main reasons why this study was conducted was to try and identify predictors for SICU admission, particularly in those patients who had unforeseen or unplanned SICU admissions.

Patient frailty was another risk factor predictive of SICU admission identified in this study. Frailty was defined as an OFS score greater than or equal to 2. Unlike some of the other variables evaluated in this study (such as uCCI), the OFS is comprised of five simple binary variables, including institutionalization, non-independent functional status, and a history of malignancy, and is relatively easy to calculate. Numerous frailty indices have been shown to be good prognostic indicators for postoperative morbidity and mortality in geriatric hip fracture patients, but only a handful of studies have investigated frailty as a predictor of SICU admission in this patient population [34]. Furthermore, the majority of previous frailty indices were not designed or validated specifically for use in geriatric hip fracture patients. To date, our study is the first to utilize patient frailty as defined by the OFS for predicting SICU admission specifically in geriatric hip fracture patients. The results arising from the prediction model are not surprising-frail hip fracture patients tend to have higher mortality and postoperative complications, and as such, are more likely to require perioperative SICU admission. Overall, frailty is an important measure to consider during the preoperative assessment of geriatric hip fracture patients, particularly because two different hip fracture patients with the same age, comorbid conditions, and even initial laboratory values could have entirely different postoperative outcomes as a result of differences in frailty [35].

Time from injury to ED presentation was the final SICU admission predictor identified in this study. This temporal variable was an interesting choice to investigate in this study for two major reasons. First, it measured the time from injury to first presentation in the ED; essentially looking at the leading time from when the injury first occurred to when the patient was first evaluated in a healthcare facility. Second, it loosely served as a measure of time during which a patient with a hip fracture did not receive medical attention in a tertiary care facility. Temporal variables such as wait times before admission or boarding time before admission are difficult to collect and interpret when it comes to predictive models. Furthermore, many previous studies have investigated differences in immediate versus delayed surgical intervention from the time of hospital admission, not necessarily time from injury-to-admission or injury-to-surgical intervention. The delays from injury to presentation in ED in a non-polytraumatized population where falls were the majority mechanism of injury (MOI) (98.4%) may reflect social determinants of health (SDoH) such as living alone without social support to find patients and get them to medical care, lower socioeconomic status and cannot afford a life-alert system or cell phone to call for help on their own, or living in an area with delayed ambulance response time for lower level triage calls due to lack of emergency medical services (EMS) responders or insufficient number of ambulances [36,37]. 

Some investigative studies and meta-analyses have demonstrated that postoperative mortality (30-day and one year) was lower in patients who underwent surgery within 24-48 hours after hospital admission [37-39]. Interestingly, there have also been multiple different studies that have concluded that there is no difference in mortality outcomes for geriatric hip fracture patients who underwent delayed surgical intervention [40-44]. The investigators in these studies argued that they found no difference in mortality outcomes for delayed surgical intervention once correcting for confounders such as age, dementia, and chronic illnesses/comorbidities. Furthermore, some even argued that medical stabilization of chronic medical conditions tended to be the cause for surgical delay in the first place and that chronic comorbidities were the culprits for negative outcomes and not the delay in surgical intervention itself.

Considering these previous findings, we postulate that time from injury to ED presentation greater than 12 hours was a predictor for SICU admission not because it delayed surgical intervention, but because it was a marker of the prolonged time during which a hip fracture patient did not receive medical attention for stabilization or optimization of comorbidities prior to surgical intervention.

Planned versus unplanned SICU admission

Patients with unplanned SICU admission had only one additional risk factor when compared to the pooled SICU admission group. This result is not entirely surprising given that the majority of the SICU admissions in the pooled group were unplanned SICU admissions (74%).

The predictors identified for patients in the planned SICU admission group were very different and did not have any commonality with the predictors for unplanned SICU admission. Some of these predictors were not entirely unexpected, however, particularly because some of the variables identified are clearly indicative of critically ill patients necessitating admission to the SICU. Those patients with a planned SICU admission included in this study had been designated for SICU admission a priori, meaning they were going to be admitted to the SICU from the start regardless of risk factors; likely because they presented to the ED in a critically ill state (possibly with cardiopulmonary or respiratory failure) necessitating SICU admission. Systolic BP < 100 and heart rate (HR) > 100 are both indicative of shock, a common criterion for SICU admission and not unexpected in critically ill trauma patients. Geriatric patients with an ISS >=15 are considered to have major trauma, and elevated scores would be expected in critically ill patients necessitating SICU admission [45]. Hyponatremia (serum sodium < 135) is the most common laboratory abnormality in hospitalized geriatric patients; again, it is not unexpected in critically ill hip fracture patients. Ultimately, the purpose of this study was to identify predictors to risk stratify those patients who might require unplanned SICU admission. Since none of the predictors in the planned SICU group had commonality with the overall (pooled) SICU admission predictors, the utility of investigating these predictors in future studies remains low.

Limitations

Although the three risk factors predicting SICU admission identified in this study show promise as useful tools to be used in risk-stratifying hip fracture patients in a clinical setting, there are some limitations that merit discussion at this time. First, the study has a retrospective design, and although it identified important predictors for ICU admission, a prospective validation study might be useful in determining the clinical applications of these predictors (i.e., creating a risk-stratifying clinical score to determine if the real-world application of the score can lead to changes in morbidity, mortality, LOS, or other outcomes in hip fracture patients). Additionally, including other variables in the predictive model, for example, type of anesthesia, may improve risk modeling of SICU admission in a geriatric population that may have respiratory disease comorbidity. The next step in our research involves the creation of a simple clinical tool and testing its predictive ability for SICU admission in a national cohort of geriatric hip fracture patients with falling as the primary MOI which was reflected in our population and is the primary cause of hip fractures (95%) in the United States [46]. Furthermore, simply identifying which patients had risk factors predictive of ICU admission does not imply that any interventions (such as early recognition, resource allocation, or targeted treatment approaches) will have any effect on important outcomes such as morbidity, mortality, LOS, or even overall cost. The answers to those questions merit separate research investigations in their own right, and those are some of the follow-up projects that have arisen out of this research study at our institution.

## Conclusions

The findings in this study are important because they identify important predictors for subsequent Intensive Care Unit (ICU) admission in geriatric hip fracture patients. Additional research is underway for developing a comprehensive yet simple risk stratification tool to aid physicians in determining which hip fracture patients are at increased risk for ICU stay in order to implement targeted treatment approaches to improve morbidity and mortality outcomes.
